# Short-Acting Testosterone: More Physiologic?

**DOI:** 10.3389/fendo.2020.572465

**Published:** 2020-09-30

**Authors:** Gerwin Westfield, Ursula B. Kaiser, Dolores J. Lamb, Ranjith Ramasamy

**Affiliations:** ^1^Aytu BioScience, Englewood, CO, United States; ^2^Division of Endocrinology, Diabetes and Hypertension, Brigham and Woman's Hospital, Harvard Medical School, Boston, MA, United States; ^3^James Buchanan Brady Foundation Department of Urology, Center for Reproductive Genomics, Englander Institute for Precision Medicine, New York, NY, United States; ^4^Department of Urology, Miller School of Medicine, University of Miami, Miami, FL, United States

**Keywords:** hypogonadism, testosterone, fertility, HPG axis, androgens

## Introduction

Pulsatile secretion of a hormone refers to the intermittent secretion of the hormone in a burst-like or episodic manner rather than constantly, with the frequency varying from minutes to hours, determined in part by the half-life of the hormone. In men, communication between the hypothalamus, anterior pituitary, and testes via the hypothalamic-pituitary-gonadal (HPG) axis regulates testosterone (T) secretion, with a negative feedback mechanism controlling circulating levels of T (1). Pulsatile release of gonadotropin-releasing hormone (GnRH) from hypothalamic neurons signals the pulsatile secretion of luteinizing hormone (LH) from the pituitary. Circulating LH, in turn, signals the testes to produce T. Once the appropriate threshold of circulating T levels is achieved, T acts as a negative feedback molecule, signaling to the hypothalamus and pituitary to inhibit secretion of GnRH and LH, respectively (2). T levels in a healthy male follow a diurnal variation and circadian rhythm, with levels highest early in the morning and subsequently declining as the day progresses, as a direct result of pulsatile LH secretion (3).

T deficiency (TD) is a condition in which insufficient T levels are attained with one or more symptoms such as fatigue or low energy, depression, low libido, erectile dysfunction, and/or poor concentration (4). The Endocrine Society recently published treatment guidelines for men with symptomatic TD, which recommend a treatment goal of achieving steady-state serum total T concentrations in the mid-normal range (5). A sustained steady-state level of T, however, differs from the normal circadian physiology of a healthy individual. The most common hormone treatment for male hypogonadism is an intramuscular (IM) injectable generic testosterone cypionate/enanthate (72.8%), with other therapies including topical gel (25%), transdermal patch (1.2%), long-acting IM injections of testosterone undecanoate (0.18%), and nasal testosterone (0.13%) (6).

## Testosterone Pharmacokinetics

The various testosterone formulations have a wide range of dosing intervals including long-acting preparations: subcutaneous pellets (3 to 6 months), injectable IM testosterone undecanoate (10 weeks); intermediate-acting preparations: IM testosterone cypionate/enanthate (1 to 3 weeks); daily preparations: topical/transdermal formulations; and short-acting preparations: oral testosterone undecanoate (twice daily) and nasal testosterone (two to three times daily). All formulations, with the exception of the short-acting ones, have a target of long-term maintenance of sustained steady state testosterone levels in the mid-normal range, which leads to suppression of endogenous activity of the HPG axis.

As previously noted, testosterone levels in young healthy males follow a circadian rhythm. T levels are highest in the morning, and lower in the evening hours. There is significant change within a 24-h period. Testosterone itself acts as a negative feedback molecule to the hypothalamus and anterior pituitary. When T levels are high enough, they signal to reduce GnRH, LH, and FSH secretion, thereby also reducing endogenous testosterone production. This occurs regardless of whether the circulating testosterone is endogenous or exogenous. If high levels of testosterone are given exogenously for extended periods of time, this can result in negative feedback to the hypothalamus and anterior pituitary, disrupting normal HPG regulation.

A pharmacokinetic study evaluating serum T levels of subjects receiving IM testosterone cypionate administration revealed supraphysiologic T levels (~1100 ng/dL) within 2–3 days after injection, whereas toward the end of the dosing interval, T levels approached subtherapeutic levels (7). Topical gel formulations achieve a sustained mid-normal T level with a once daily application (8). While the topical gel results in less fluctuation of T levels between dosing intervals when compared to IM T, the sustained T levels result in inhibition of HPG axis activity (9). The inhibition of HPG axis activity is evidenced by the nearly full suppression of gonadotropin levels following treatment with either IM injectable testosterone (10) or topical gel administration (9). Nasal administration of T (4.5% testosterone nasal gel, Natesto) allows for rapid absorption through the nasal mucosa such that serum T levels reach a peak concentration in ~40 min. Once in the circulation, the T is quickly metabolized, with a return to near baseline T levels in 3–6 h (11). Therefore, multiple administrations of nasal T throughout the day (three times daily) maintain normal mean serum T levels over 24 h. The fluctuations in T levels potentially minimize the duration of exposure to exogenous T that is suppressive to the HPG axis, compared to other available T therapies.

## Testosterone, HPG Axis, and Gonadotropins

When discussing T therapy with patients, fertility, and family planning are important considerations, because treatment with injectable and topical T products increases the risk of oligo- or azoospermia and infertility (12). Treatment with exogenous T that maintains serum T levels in the mid-normal range suppresses the pituitary gonadotropins, LH and follicle-stimulating hormone (FSH), to nearly undetectable levels, resulting in impaired sperm production (10).

Data from the Phase 3 clinical study for nasal testosterone showed minimal reduction in mean LH and FSH levels while restoring normal T levels in hypogonadal men (13). Studies in mice support the hypothesis that longer-acting T has a more suppressive impact on fertility when compared to short-acting T (14). A recent Phase 4 clinical study reported that nasal T restored normal T levels in hypogonadal men, improved hypogonadal symptoms, preserved serum LH and FSH levels, and also preserved mean semen parameters, including sperm concentration, sperm motility, and total motile sperm count (TMSC) in 90–95% of men (15). With weekly injections of testosterone enanthate, ~65% of men became azoospermic after 6 months of therapy (12). However, after 6 months of nasal T treatment, semen parameters remained statistically unchanged from baseline. It appears that the pharmacokinetics and short-acting nature of the nasal T preparation results in minimal suppression of the HPG axis, compared to longer-acting forms of T. Treatment with an FDA approved short-acting testosterone has the potential to treat hypogonadal symptoms while better preserving fertility compared to currently available longer-acting modalities.

## Side Effects with Testosterone Therapies

Some, but not all, side effects from testosterone therapy (TTh) include reduced sperm production or azoospermia as described above, polycythemia, and inhibition of the HPG axis. Prolonged exogenous T administration using injections, pellets, and topical gels result in varying degrees of these common side effects. On average, long-term topical gel TTh leads to an increase in hematocrit of 1–2%. Injectable TTh leads to an average increase in hematocrit of 2–4% (16). In contrast, after one year of therapy with nasal TTh, there was no significant increase in hematocrit (17).

## Warnings and Precautions with Testosterone Therapies

In addition to the commonly reported side effects with TTh, there are a number of FDA mandated black box warnings associated with T formulations. Long-acting IM testosterone undecanoate requires an in-office visit due to its warning for serious pulmonary oil microembolism (POME) reactions and anaphylaxis (18). Topical T gel formulations come with a black box warning for risk of transference and secondary exposure to T (8). Two recently approved formulations—a subcutaneous auto-injection of testosterone enanthate (19), and oral testosterone undecanoate capsules (20)—come with a black box warning for blood pressure increases. The short-acting nasal testosterone gel has no black box warnings, including no risk of secondary transference or increase in blood pressure (21). Nevertheless, what is needed is a head-to-head study comparing outcomes (T levels, blood pressure, hematocrit) among the different formulations in order to answer the question of safety and efficacy.

## Final Thoughts

**Figure 1:**
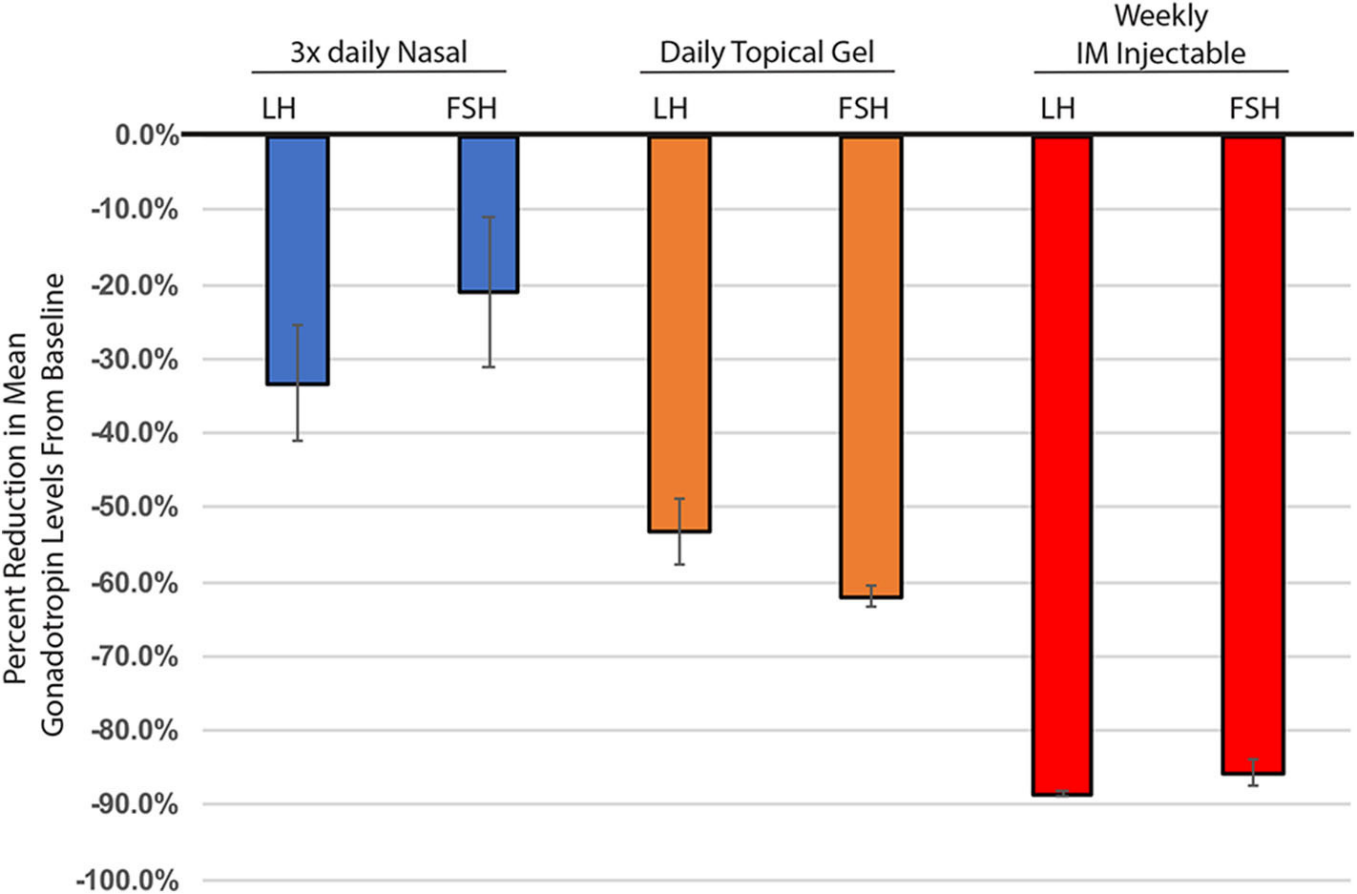
Percent change in mean gonadotropin levels (LH & FSH), from baseline through 6 months of testosterone treatment. Nasal testosterone (blue), dosed t.i.d., adapted from (15), *n* = 33. Topical testosterone (orange), dosed daily, adapted from (9), *n* = 123. IM injectable - 100 mg testosterone enanthate, (red), adapted from (10), *n* = 10. All changes from baseline were statistically significant. Nasal testosterone—FSH *p* = 0.03, all others *p* < 0.001. Standard error calculated using delta method.

Endocrine systems are regulated dynamically in response to positive or negative stimuli within a homeostatic environment. Modalities of T therapy evolved to extend the dosing interval and maintain sustained “steady-state” T levels. Long-acting TTh can inhibit the HPG axis, which in turn suppresses pituitary LH and FSH secretion, reducing circulating levels of LH and FSH and endogenous T production (Figure 1). These endocrine changes result in suppression of spermatogenesis and infertility, as well as having other side effects. The detrimental impact on sperm production from these long-acting testosterone therapies led to the use of many off-label products. The use of clomiphene citrate to stimulate gonadotropin production and subsequently increase testosterone levels is used to treat hypogonadism, while also trying to preserve fertility.

Short-acting T therapy, consisting of several doses of T with a shorter half-life throughout the day, minimizes inhibition of the HPG axis and reduces the impairment of spermatogenesis. Utilizing FDA approved, shorter acting forms of T therapy to maintain homeostasis that more closely reflects normal physiology offers great promise for the treatment of men with hypogonadism—an advantage over long-acting formulations. This therapy would allow use of FDA approved testosterone supplementation exogenously as opposed to using off-label treatment strategies, while also preserving what fertility the treated subject has. Support of this hypothesis is needed from additional, longer duration studies utilizing short-acting testosterone. However, it is encouraging that studies performed to date tend to support the hypothesis. The literature is well established that long-acting testosterone results in HPG suppression and resulting changes in physiology. Short-acting T therapy may be paradigm-changing for the treatment of T deficiency.

## Author Contributions

GW wrote the initial draft which was revised, edited, and expanded by UK, DL, and RR. All authors contributed to the article and approved the submitted version.

## Conflict of Interest

GW is an employee of Aytu BioScience Inc., UK and RR are consultants for Aytu BioScience Inc. DL serves on the scientific advisory board of Celmatix, with no financial compensation and is Secretary-Treasurer of the American Board of Bioanalysts. The reviewer JS declared a past collaboration with one of the authors DL to the handling editor.
